# Automated workflows for modelling chemical fate, kinetics and toxicity

**DOI:** 10.1016/j.tiv.2017.03.004

**Published:** 2017-12

**Authors:** J.V. Sala Benito, Alicia Paini, Andrea-Nicole Richarz, Thorsten Meinl, Michael R. Berthold, Mark T.D. Cronin, Andrew P. Worth

**Affiliations:** a Chemical Safety and Alternative Methods Unit, EURL ECVAM, Directorate F – Health, Consumers and Reference Materials, Joint Research Centre, European Commission, Ispra, Italy; b Liverpool John Moores University, School of Pharmacy and Biomolecular Sciences, Byrom Street, Liverpool L3 3AF, UK; c KNIME.com AG, Zurich, Switzerland; d Universität Konstanz, Fachbereich Informatik und Informationswissenschaft, Box 712, 78457 Konstanz, Germany

**Keywords:** KNIME, Virtual cell based assay, Physiologically based kinetic models, PBPK, Toxicity, Automated workflows

## Abstract

Automation is universal in today's society, from operating equipment such as machinery, in factory processes, to self-parking automobile systems. While these examples show the efficiency and effectiveness of automated mechanical processes, automated procedures that support the chemical risk assessment process are still in their infancy. Future human safety assessments will rely increasingly on the use of automated models, such as physiologically based kinetic (PBK) and dynamic models and the virtual cell based assay (VCBA). These biologically-based models will be coupled with chemistry-based prediction models that also automate the generation of key input parameters such as physicochemical properties. The development of automated software tools is an important step in harmonising and expediting the chemical safety assessment process. In this study, we illustrate how the KNIME Analytics Platform can be used to provide a user-friendly graphical interface for these biokinetic models, such as PBK models and VCBA, which simulates the fate of chemicals in vivo within the body and in vitro test systems respectively.

## Highlights

• The VCBA is a mathematical model that simulates in vitro fate of chemicals and the corresponding cellular effect.
• The VCBA has been implemented in an open access web-based KNIME platform for ease of use.
• KNIME Analytics Platform can be used to provide a user-friendly graphical interface for biokinetic models.

## 1 Introduction

Future human safety assessments will rely increasingly on the use of multi-scale models, such as physiologically-based kinetic/dynamic (PBK/D) models and virtual cell based assay (VCBA) models to calculate internal concentrations and perform extrapolations such as in vitro to in vivo extrapolation (IVIVE). These biologically-based models will be coupled with chemistry-based prediction models that also automate the generation of key input parameters such as physicochemical properties. The implementation and integration of such models within an automated and user-friendly computational platform will make them more easily accessible and applicable to support the chemical risk assessment process.

The development of computational tools and predictive models to support the safety assessment of chemicals, in particular cosmetics-related substances, was the goal of the EU COSMOS project (http://www.cosmostox.eu/), with the aim to making the models publicly available in a user-friendly format. The models included prediction models for specific effects based on structure-activity relationships, absorption models to support extrapolation from oral to dermal exposure, especially relevant for cosmetics substances, as well as the biokinetics models described here.

The VCBA can be used to support (i) the design of in vitro High Throughput Screening (HTS) experiments; (ii) hazard identification (based on acute systemic toxicity); and (iii) QIVIVE approaches to help risk assessment decision making. The VCBA is a mathematical model that was built using the R language and was applied to study the toxicological effects of chemicals on cells (Zaldívar et al., 2010, Zaldívar et al., 2012, Zaldívar Comenges et al., 2011, Zaldívar Comenges et al., in press). The model consists of differential equations whose solution allows the calculation over time of the dissolved concentration of a chemical in a well plate as well as the internal concentration in the cells. The VCBA model (Zaldívar Comenges et al., in press) consists of four inter connected models, describing (i) a fate and transport of the time-dependent chemical concentration in the medium and in the headspace; (ii) partitioning in the cell, assuming an instant partitioning of the chemical to water, lipid, and protein within the cell after uptake; (iii) the four stage of cell cycle, (cell growth and division); and (iv) cell dynamics: toxicity and effect. In addition the model takes into account the experimental conditions (i.e. well shape).

Similarly, PBK models mimic the distribution of a chemical in the body. These models represent the body as interconnected compartments, describing the organs, and with inter-compartment fluxes described by differential equations. The level of complexity of these models depends on the intended application and available biological information: by predicting concentration and time profile curves (C_max_ and AUC), these models can support the submission of drugs to be evaluated by medical agencies; they can be used to support chemical risk assessments; as well as informing experimental design. In the last decade there has been an increase in development of PBK models, because of more accurate simulations of in vivo adsorption, distribution, metabolism and excretion processes compared to the classical PK models (Gajewska et al., 2015). For example, 11 chemical specific PBK models were developed within the EU COSMOS project (Bois et al., in press, Teng et al., 2015, Gajewska et al., 2014, Gajewska et al., 2015). Furthermore, an initiative to develop a database of existing PBK models is currently ongoing at US EPA (Lu et al., 2016). This database provides useful information on how the models were built. Furthermore, several commercial tools are available to program (Matlab, R language, Berkeley Madonna, etc.), build and use (SimCyp, gastroplus/ADMET, PkSim, ACSL/X, etc.) as well as tools that can be used without a commercial licence: MERLIN EXPO (Ciffroy et al., 2016, Suciu et al., 2016), MEgen and Rvis, (Loizou and Hogg, 2011) COSMOS (Bois et al., in press).

The coupling of a PBK model, describing the kinetics, and the VCBA, describing the dynamic effects (that can be adverse or beneficial) will give rise to a model that can be applied to extrapolate from an in vitro concentration to the external dose of exposure. In vitro to in vivo extrapolation (IVIVE) is a useful approach in the prioritization of chemical testing in chemical risk assessment (Kramer et al., 2015, Allen et al., 2017). To better exploit the utility of IVIVE, there is a need to develop automated tools that perform the extrapolation in a simple and fast way.

**Fig. 1:**
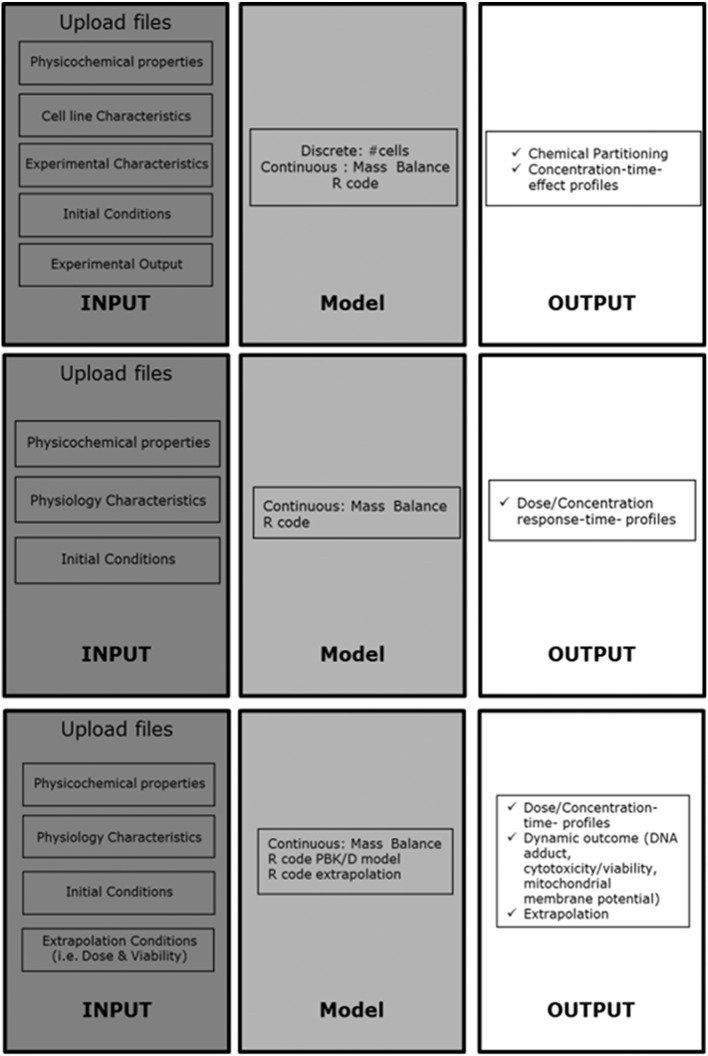
A. Schematic representation of the general setup of the automated VCBA KNIME workflow for the kinetic models developed; this set up is based on three zones: input, model (core zone where the code is kept), and output. B. Schematic representation of the general setup of the automated physiologically based kinetic model; C. Schematic representation of the general setup of the automated in vitro to in vivo extrapolation approach.

As described above, these models consist of many parameters that are feeding the equations, physicochemical, cell line and experimental characteristics. The use of such models entails a need to manage a large and diverse set of data, from parameters to in vitro dose response curves analysis. The best way is to implement good data management practice and develop automated workflows. The KNIME Analytics Platform (Berthold et al., 2009; http://www.knime.org/) is a free, user-friendly graphical workbench for data analytics including data management, data transformation, investigation, visualization and reporting. The COSMOS biokinetic models have been implemented in two KNIME versions, so that they can be executed either locally on a desktop computer (following installation of the KNIME Analytics Platform) or directly online from a web browser using a KNIME WebPortal (http://knimewebportal.cosmostox.eu). With a view to promoting end-user acceptance of these KNIME-based workflows, bespoke documentation is available (see http://www.cosmostox.eu/what/webtutorials/). Fig. 1 describes schematically how the workflows for the VCBA, PBK models, and IVIVE approach were set up, with an input zone indicating which input parameters are needed to run the simulation per type of workflow, the core model and output zone, with description of which output the model provides per workflow. The optimal interface should allow untrained users to use these workflows in an intuitive way. In this study, we illustrate how the KNIME workflows for the PBK model, the VCBA, and IVIVE approach were built and how they can be used as a user-friendly graphical interface to predict different model simulations and outcomes. The development of automated software tools is an important step in harmonising and expediting the chemical safety assessment process.

## 2 Methodology

We implemented the PBK models, VCBA, and the IVIVE approaches as open source platforms using KNIME (version 3.0) and R programs (which both are freely available). The KNIME Analytics Platform is a user-friendly graphical workbench for data analysis (http://www.knime.org/) and R is a language and environment for statistical computing and graphics (http://www.r-project.org/). KNIME consists of a series of pieces of program code called nodes that can be connected in such way that the input of one node is the output of the previous one. Each node has a dialog in which the user can configure the operation of the node.

### 2.1 Implementation of the virtual cell-based assay model in KNIME

This VCBA KNIME implementation includes three important features:

• Several operational modes: single exposure, repeated exposure, parameter optimization and optimization check. These are further described below.
• Three separate zones: input, model, and output.
• Can be run on the KNIME Server (online) or in the KNIME Analytics Platform as locally installed version in a desktop computer.

**Fig. 2:**
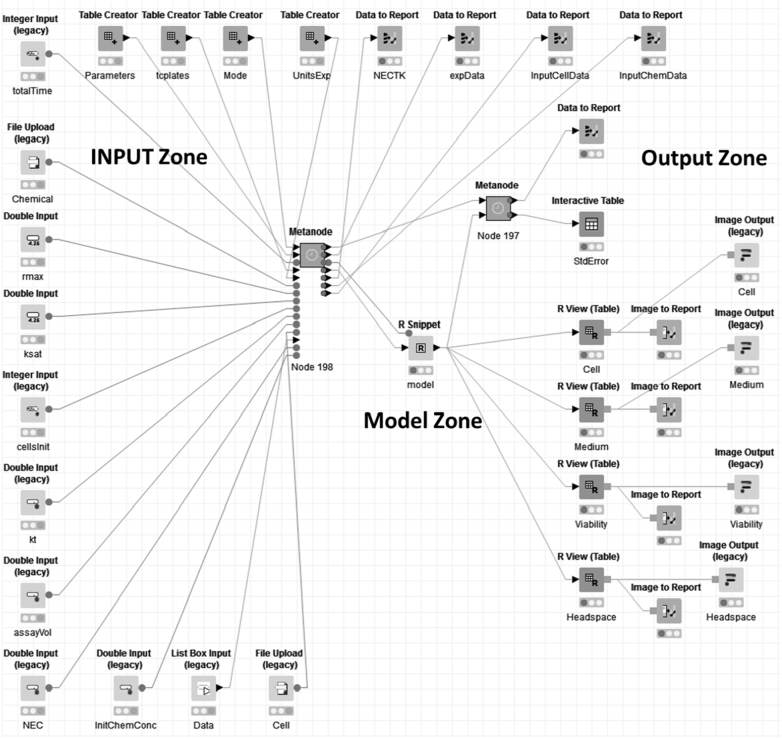
KNIME workflow of the virtual cell based assay model, divided into three zones: Input Zone, Model Zone (where the model script is kept and executed), and the Output Zone. For purposes of figure presentation the nodes that where not relevant for running the workflow, were collapsed into metanodes.

In the following, we will introduce and explain the most significant nodes that build up the KNIME workflow as represented in Fig. 2.

The automated VCBA KNIME workflow can be run in several modes. The *Single Exposure* mode of operation describes the process for a single exposure of the chemical to the cells. The present version can be run for several chemical concentrations. From one run to the next all parameters are reset to initial values except the initial chemical concentration. The other mode is *Repeated Exposure*, which describes the effect of several cell exposures of a single chemical concentration. From one run to the next, the last values of chemical concentration inside the cell and the number of cells are considered as the initial values for the next exposure, whereas the initial chemical concentration remains the same. The toxicological effect of cell exposure to chemicals in the VCBA is described by two parameters: the No Effect Concentration (NEC) and kt (where t is exposure time, and k is a characteristic of the chemical and cell line). The values of NEC and kt are then optimised by comparing the simulated (*Viability*_*sim*_) and experimental (*Viability*_*exp*_) cell viabilities and by minimizing the following error function:

(1) $$\mathrm{error}=\sum_{i=1}^{n^{o}\exp}{\left({\mathrm{Viability}}_{\exp}-{\mathrm{Viability}}_{\mathrm{sim}}\right)}^{2}$$

The *optimization check* gives us a graphical interface which is used to validate the values of NEC and kt. Paired values of chemical concentration and viability are introduced in a *List Box Input* node, while the initial values of NEC and kt are in two *Double Input* nodes. The Optimization Check functionality was separated from the Parameter Optimization to enable available values of NEC and kt to be entered directly without the need to run the time-consuming Parameter Optimization Mode every time.

The building blocks of the three zones are explained in more detail in the following section.

1) The **Input Zone** is used to feed the model with the necessary input data. It consists of a series of nodes where files are uploaded to input and select data for chemicals and cell lines:

• Data related to the cell line such as duration, mortality, mass, volume in each cell stage, cell duration (see Zaldívar Comenges et al., in press).
• Data related to the organic compound. By uploading a file containing the information on the parameters for the organic compound to be run. The chemical data needed to run simulation are chemical name (Chem name), CAS number (cas), logKow, molar volume, atomic diffusion, MW, water degradation, air degradation, and Henry's law constant (see Zaldívar Comenges et al., in press).
• Toxicological data: the NEC and kt for each chemical and cell line (see Zaldívar Comenges et al., in press).
• Experimental set-up data: The Tissue Culture (TC) plates node is a table that contains information on the technical specifications of the plates in the High Throughput Screening (HTS) as well as non-HTS experiments. These data are used to compute properties related to fate and transport, such as chemical binding to plastic or evaporation across the air-water interface (see Zaldívar Comenges et al., in press).
• Data to be simulated (e.g. range of chemical concentration inside the cells, time of simulation).

For data to be simulated, the data introduced depends of the mode of operation:

• Single Exposure: Several chemical concentrations to be simulated are introduced in the *List Box Input* node separated by “,”.
• Repeated Exposure: Unique chemical conc. in a *Double Input* node, the interval between doses and the number of repeated exposures in an *Integer Input* node.
• Parameter Optimization: Is used to optimize values related with toxicological data: NEC and kt. In a *List Box Input* node pairs of chemical concentration and viability are introduced and in two *Double Input* nodes, the initial values of NEC and kt. In the case of single and repeated exposure the values of NEC and kt are in the toxicological data table.

To run the model, the following additional information is needed: Total time (*Integer Input* node), Initial number of cells (*Integer Input* node), Assay Volume (*Double Input* node).

**Fig. 3:**
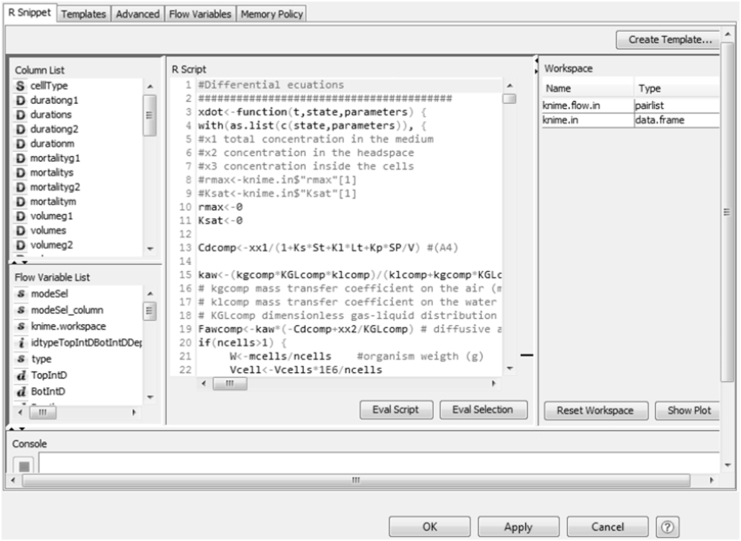
Detail of R script code of the VCBA model (R snippet node).

2) The **Model Zone** contains the mathematical script (Fig. 3) and executes the simulations. The input parameters are provided to the R Snippet node through the variable *knime in*. We have written the differential equations describing the VCBA in R language and have integrated them into KNIME through its *R* integration. In this way the model is accessible to the user without the need to modify it. The differential equations describing the mass balance resulting from fate, cell dynamics and toxicodynamics are solved by the *DeSolve R package* (see Zaldívar Comenges et al., in press, Paini et al., 2016). The VCBA is an integrated mathematical model that can be run in several operational modes (which can be *Single Exposure simulation*, *Repeated Exposure simulation*, *Parameter Optimization*, *Optimization Check*). To perform this, we create a function called *coreModel* inside the R snippet node. This function is an R function that has five input parameters: chemical concentration in the medium, chemical concentration inside the cell, the number of cells, NEC and kt. The *coreModel* function is called several times depending on the operation mode. In the case of single exposure, *coreModel* is called one time for a single chemical concentration to be simulated whereas the rest of parameters remain constant. In the case of repeated exposure, there is a single chemical concentration exposure and the chemical concentration inside the cell and the resulting number of cells is taken into consideration for the next exposure. In the Parameter Optimization mode, the core model calculates the simulated viability for each chemical concentration based on the initial values of NEC and kt given in the two *Double Input* nodes. The results of the R Snippet node are output using the *knime out* variable.

3) **Output Zone**: The output from the R Snippet node is sent to several output nodes to provide and visualize the results. For tabulated data results, KNIME offers a variety of nodes that can store data. For graphical results, *R Table View* node is a KNIME node that generates graphics using R code. To show the results in a KNIME Server a series of additional nodes are needed. For graphics, each *R Table View* node is associated with an image output node. For small tables, a *Textarea Output* node can be used inserting the flow variable values into a structured html table. Furthermore, two *report* nodes provide a downloadable report of the results.

### 2.2 Implementation of the physiologically based kinetic models in KNIME

The PBK model KNIME workflow is set up in a similar way as the VCBA one, with an Input, Model and Output zone. The input parameters, physiological data and physicochemical parameters are stored in a file and upload in the node. The range of time and doses (initial and final) must be provided. The R code representing the PBK model is stored in the model zone of the workflow and the output is reported as graphical representation of concentration time profile curves per target organ and as a table to be able to extract AUC and C_max_ values (Fig. SM1). To describe different exposure we used the same workflow with two different R codes describing oral and dermal exposures, respectively (Fig. SM2).

### 2.3 Implementation of the extrapolation from in vitro to in vivo in KNIME

The KNIME workflow built for extrapolating from an in vitro concentration to an in vivo exposure doses (IVIVE KNIME workflow) was built in three parts, input, model, output zone (Fig. SM3). The input parameters are the same as reported for the PBK model (physiological and physicochemical properties) with addition of the cell model (thus cell type properties) and toxicity effect (No Effect Concentration, NEC, and Killing rate, Kr), so to be able to simulate dynamic effect (i.e. viability). The present workflow included three R codes, the first one representing the PBK model, the second one built to allow extrapolation of the number of DNA adducts formed and the third one to allow the extrapolation from a range of concentrations simulated to a range of exposure doses and to link to the effect based on the output of the first R code (PBK model). The extrapolation was done using the dose response curve obtained by the PBK model simulation. So far these workflows are chemical specific, and only 3 workflows were built for estragole, caffeine (Gajewska et al., 2015), and coumarin.

### 2.4 Implementation as COSMOS KNIME WebPortal version

The COSMOS KNIME WebPortal is freely accessible (http://knimewebportal.cosmostox.eu) and is supported by COSMOS Space (http://cosmosspace.cosmostox.eu), a facility to share predictive toxicology resources. The COSMOS Space provides free registration to login to the COSMOS KNIME WebPortal (log-in credentials are the same as for COSMOS Space) and hosts the workflow documentation as well as user guidance. Web tutorials for the different workflows are available from the COSMOS project website (http://www.cosmostox.eu/what/webtutorials/).

The PBK models, VCBA and the IVIVE approach workflows have been implemented additionally as WebPortal version. This allows execution of workflows through a web interface from any recent web browser by access to the KNIME Server, without installation of the software locally and without knowledge of the KNIME workflows as such. The WebPortal allows for a step-by-step execution. Each step asks for user input, such as files or model parameters, potentially also dependent on previous inputs. After all inputs have been provided, the workflow is executed and the results can be downloaded as files and/or graphical reports are generated as summaries.

The VCBA KNIME nodes for input parameters and output data are the same in the desktop version as in the server version. Since the Parameter Optimization mode requires a long time to optimize the parameters, this can be performed only in the desktop version.

**Fig. 4:**
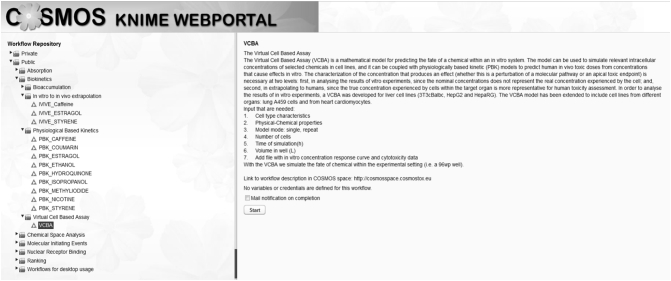
Description of the VCBA model in the COSMOS KNIME WebPortal.

The first screen in the COSMOS KNIME WebPortal is a textual introduction to the model (Fig. 4). Clicking on start a new page will appear with blank boxes where parameters can be filled in by the user. To create this form a series of KNIME nodes are used: i) *Integer Input* node; ii) *Double Input* node and so on, as described previously for the input zone.

## 3 Results

### 3.1 Application of the automated tools developed

Now that the VCBA automated tool is built and ready to use (either locally or from the WebPortal) we will show some concrete simulation results using two chemicals, acetaminophen and caffeine, as illustrative examples. The first version of the model (2015) works in single dose mode for the following compounds: acetaminophen, verapamil hydrochloride, acetylsalicylic acid, maprotiline, cycloheximide, sodium lauryl sulphate, tert-butylhydroperoxide, valproic acid, rifampin, thioridazine hydrochloride, caffeine, colchicine, acrylamide, diphenhydramine, pentachlorophenol, disopyramide, chloroquine diphosphate, tetracycline hydrochloride, amiodarone hydrochloride, carbamazepine, HPTE, pyraclostrobin, diquat dibromide, abamectin, bisphenol A, and benomyl. The VCBA currently provides repeated exposure simulations for caffeine, amiodarone, acetaminophen (for only HepaRG, Paini et al., 2016). However, in 2016 the configuration of the VCBA was improved, and presented in this work, now the model is able to run for any type of chemical and cell line types, in single and repeated exposure.

**Fig. 5:**
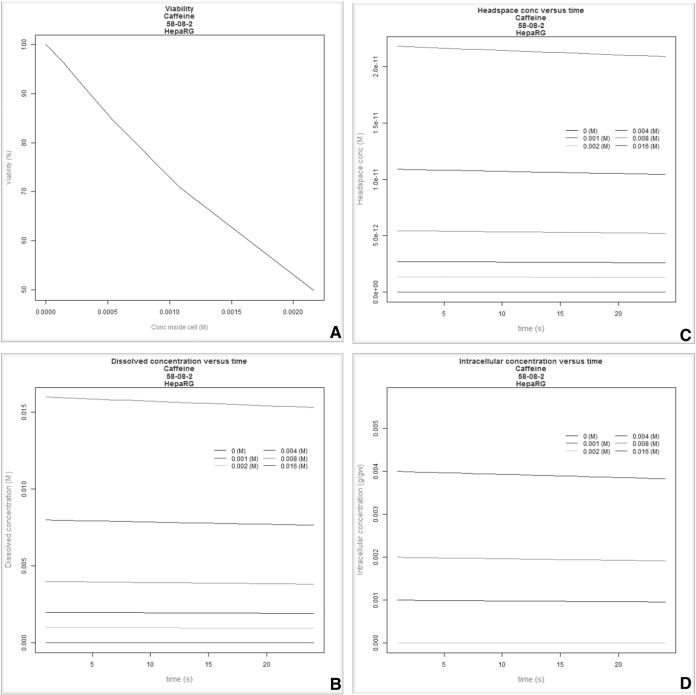
VCBA Caffeine-HepaRG simulations for single exposure. (A) Viability versus chemical concentration in the cell expressed in g/g wet weight. Concentration of caffeine in the medium (M) (B), headspace (M) (C) and inside of the cell (g/gww) (D) respectively versus time. The Legend reports the starting nominal concentrations used for simulation in M.

**Fig. 6:**
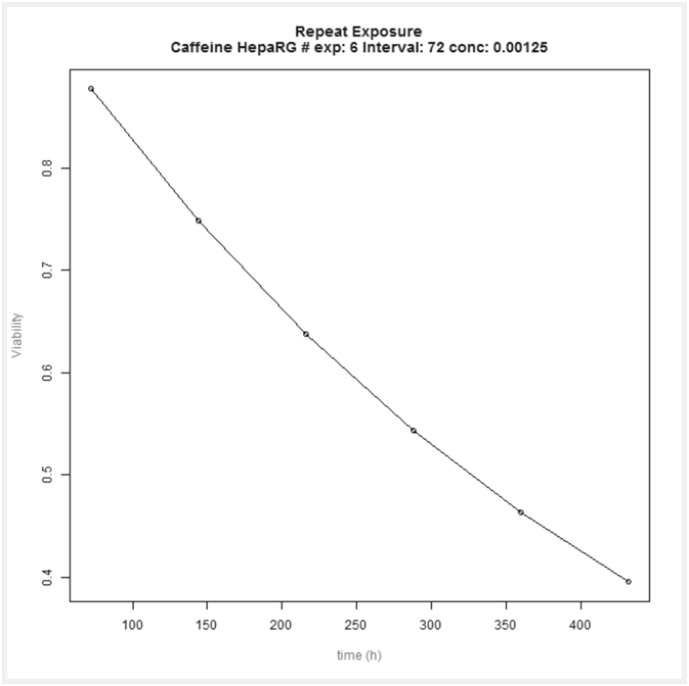
VCBA simulations of HepaRG cell viability following repeated exposure to caffeine.

**Fig. 7:**
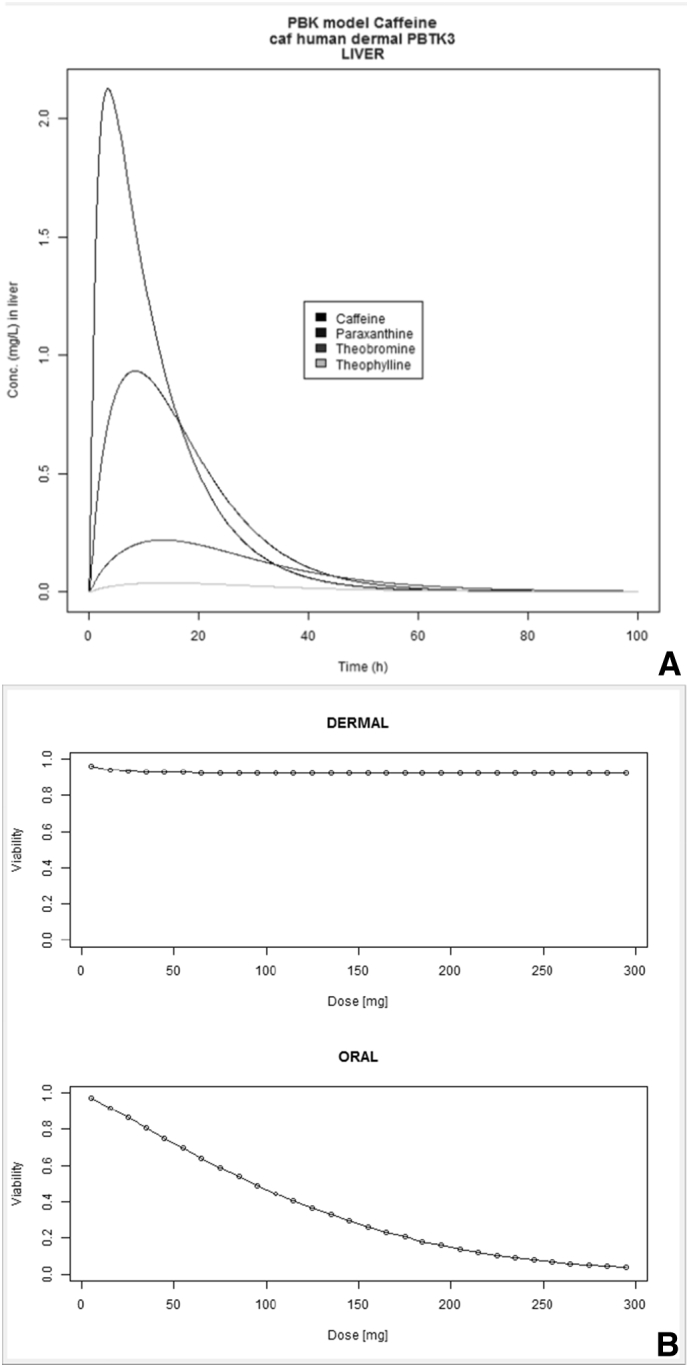
A. Concentration – time profile curves from the PBK model built for caffeine and relevant metabolites (corresponding to the PBK model workflow in Fig. 4A). B. PBK model simulation of viability – dose response for oral and dermal exposure (corresponding to the PBK model workflow in Fig. 4B).

**Fig. 8:**
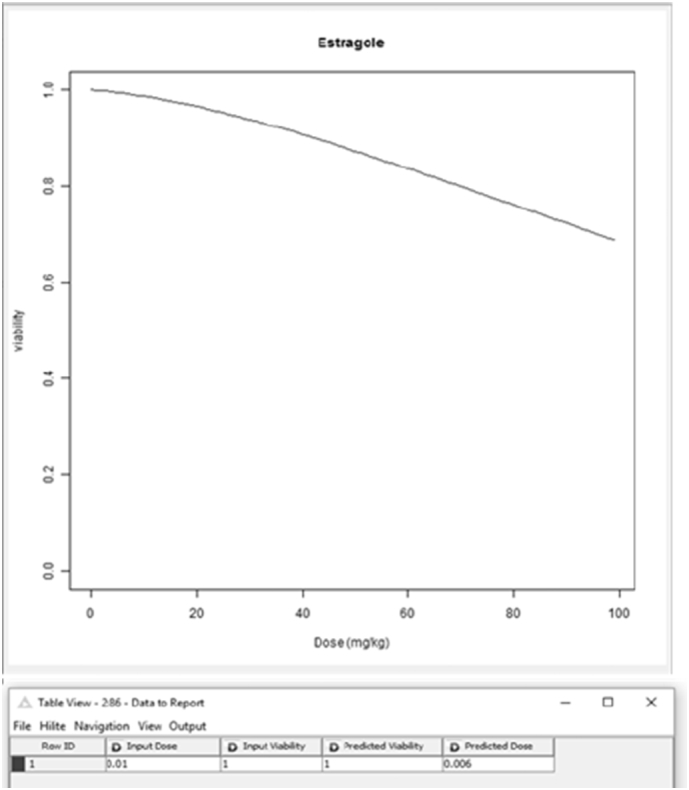
Viability - dose response curve from IVIVE workflow, including the extrapolation table from which the selected dose or viability (input) versus the corresponding predicted viability or dose, respectively (corresponding to the PBK model workflow in Fig. 5).

In Fig. 5, Fig. 6 we report the simulation results of the VCBA for a single and repeated exposure mode simulation for caffeine in HepaRG cell lines, the results of the viability versus concentration in the cell, and concentrations in the medium, headspace and inside of the cell versus time, respectively. Fig. 7A depicts concentration – time profile curves from the PBK model built for caffeine (corresponding to the PBK model workflow in Fig. SM1). Fig. 7B, shows the PBK model simulation of viability – dose response for oral and dermal exposure (corresponding to the PBK model workflow in Fig. SM2). Fig. 8 shows the viability - dose response curve simulated by the IVIVE workflow as well as the extrapolation table from which the selected dose or viability (input) can be used to predict the corresponding viability or dose, respectively (corresponding to the PBK model workflow in Fig. SM3). So far the PBK model workflows are chemical specific, and only nine (9) workflows were built, for the IVIVE approach only three (3) workflows were built for estragole, caffeine (Gajewska et al., 2015), and coumarin. In the supplementary material Fig. SM4 shows the overview of how the results, for the VCBA, are displayed in the KNIME WebPortal. These graphical representations are also available in table format for easy access to specific value, and a summary report can be downloaded in different formats (pdf, word, excel).

## 4 Discussion and conclusions

In this study, we have illustrated step by step how the PBK models, VCBA, and the IVIVE approaches were implemented in KNIME. The aim of this work was to illustrate how the PBK model and VCBA, both biologically-based mathematical models, can be implemented in KNIME, thereby providing a user-friendly tool for scientists and safety assessors. The VCBA model can be used to support the design of in vitro experiments, e.g. the choice of test concentrations and time points for endpoint measurement. In the decision-making context, the VCBA can also be combined with PBK models to support the risk assessment of chemicals, for example by carrying out in vitro in vivo extrapolation (IVIVE) of no effect (safe) exposure levels (Yoon et al., 2012, Yoon et al., 2014, Groothuis et al., 2013, Gajewska et al., 2015, Hamon et al., 2015, Heikkinen et al., 2013, Wetmore, 2015).

The characteristics of the VCBA KNIME workflow and how it can be applied to provide a user-friendly graphical interface for analysis of several outcomes was illustrated, to predict time concentration profiles, such as intracellular concentration from the VCBA. The first generation of the KNIME VCBA workflow included a drop down list reporting 35 chemicals (Paini et al., 2016), for which information was available to perform simulations; however, the newly implemented workflow can be now run for any chemical, as long as the input properties are collected in a file and uploaded thus making this model usable for different chemicals and cell line types. We are still exploring the possibilities of extending the VCBA to different biological effects, which is currently able to simulate cytotoxicity following single or repeated exposure. The PBK models were developed for nine chemicals (see Bois et al., in press) and were all implemented in KNIME. MEgen (Loizou and Hogg, 2011) a PBK model generator can be used to build more PBK models, which could then be exported as R files into KNIME workflows for ease of application. At present IVIVE workflows for extrapolation of cell viability to external doses are chemical-specific, and applicable to estragole, caffeine (Gajewska et al., 2015) and coumarin.

The VCBA and PBK models fit into a suite of predictive computational models, which have been developed in the EU COSMOS project to support the safety assessment of chemicals, and in particular cosmetics-related substances. These tools are based solely on in vitro and in silico predictions thus promoting the 3Rs approaches. The biokinetic workflows are complemented by a range of chemistry-based workflows developed within COSMOS to support the safety assessment process, with a focus on cosmetics-related substances, for which the dermal route of exposure plays a major role (Richarz et al., 2015a, Richarz et al., 2015b). Within this series of computational tools developed, the VCBA and PBK models represent the fate of a chemical in a multi-well plate and in the body, respectively. The VCBA also includes a module that simulates a dynamic effect as cell toxicity in multiple cell lines, additionally. COSMOS KNIME prediction models have also been developed for specific target organ effects such as nuclear receptor binding (Steinmetz et al., 2015). Overall, PBK models simulate relevant time profile concentrations during absorption, distribution, metabolism and excretion within the body. When coupled with in vitro dynamics, PBK models can be used to relate an external exposure dose to intracellular concentrations and target-organ levels. The majority of available in vivo toxicity data are relate to oral administration. Thus models for skin permeability and gastrointestinal absorption contribute to the extrapolation from oral to dermal exposure.

In the interests of transparency, extensibility and ease of use, the developed COSMOS models have been implemented in KNIME and made publicly available as open-source, automated tools (Richarz et al., 2015a, Richarz et al., 2015b). KNIME is a flexible interface allowing users (e.g. researchers, risk assessors) to use these models in an easy way, integrating access to databases, data processing and analysis, as well as modelling approaches into flexible computational workflows. These workflows can be run on a desktop computer (following installation of the KNIME Analytics Platform), or simply by accessing the COSMOS KNIME WebPortal (http://knimewebportal.cosmostox.eu/), without the need to install any software locally. The WebPortal allows access to the KNIME Server and execution of workflows through a web interface from any recent web browser, without knowledge of the KNIME software as such. COSMOS Space (http://cosmosspace.cosmostox.eu/) hosts the workflow documentation and user guidance, including a list of all available workflows. Web tutorials for the workflows are available at http://www.cosmostox.eu/what/webtutorials/. The description of the PBK models and the VCBA can also be found in the EURL ECVAM database, the DataBase service on Alternative Methods to animal experimentation (DB-ALM) (Method Summary no. 162). DB-ALM is a public database service that provides evaluated information on development and applications of advanced and alternative methods to animal experimentation in biomedical sciences and (regulatory) toxicology (http://ecvam-dbalm.jrc.ec.europa.eu/beta/).

In conclusion, the COSMOS biokinetic models in the COMSOS KNIME WebPortal allow for an intuitive step-by-step execution, but are restricted to certain pre-configured settings. The use of the COSMOS biokinetic model workflows in the KNIME Analytics Platform, on the other hand, gives end users more freedom in executing and refining the model parameters and input data according to their own needs. At the present time, these automated workflows cannot be used to replace the need for expert judgement within the risk assessment process. However, we anticipate that their use will not only expedite the safety assessment process, but will also ensure reproducibility and traceability in some of the key steps.

## Appendix A Supplementary data

Supplementary figures Image 1
