# Supplementary material for: Automated workflows for modelling chemical fate, kinetics and toxicity
Source: Toxicol In Vitro. 2017 Dec;45:249–57. doi: 10.1016/j.tiv.2017.03.004 (PMC5745146; doi:10.1016/j.tiv.2017.03.004)
Supplement: Supplementary file 2 — Supplementary figures [file mmc2.docx]

# Supplementary Material

# Automated workflows for modelling chemical fate, kinetics and toxicity

***José Vicente Sala Benito*** ^a^***, Alicia Paini*** ^a⃰^***, Andrea-Nicole Richarz*** ^b^***, Thorsten Meinl***^c^***, Michael R. Berthold^d^, Mark TD Cronin*** ^b^***, Andrew P Worth*** ^a^

^a^ Chemical Safety and Alternative Methods Unit, EURL ECVAM, Directorate F – Health, Consumers and Reference Materials, Joint Research Centre, European Commission, Ispra, Italy

^b^ Liverpool John Moores University, School of Pharmacy and Biomolecular Sciences, Byrom Street, Liverpool L3 3AF, UK

^c^KNIME.com AG, Zurich, Switzerland

^d^ Universität Konstanz, Fachbereich Informatik und Informationswissenschaft, Box 712, 78457 Konstanz, Germany

**** Corresponding author:*** alicia.paini@ec.europa.eu

Joint Research Centre

Via E. Fermi 2749, TP 126

I-21027 Ispra (VA), Italy

tel.+39-0332-78 3986

fax +39-0332-78 9963


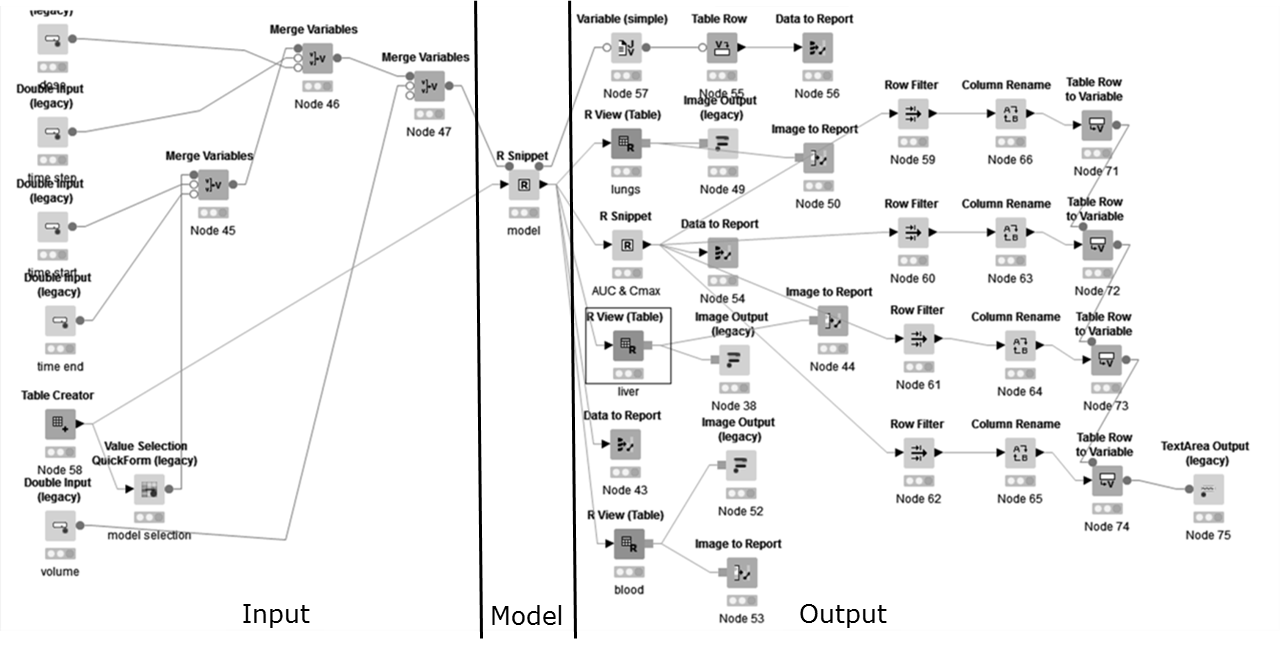


**Figure SM1**. Example of KNIME workflows: for (A) Physiologically Based Kinetic Models and


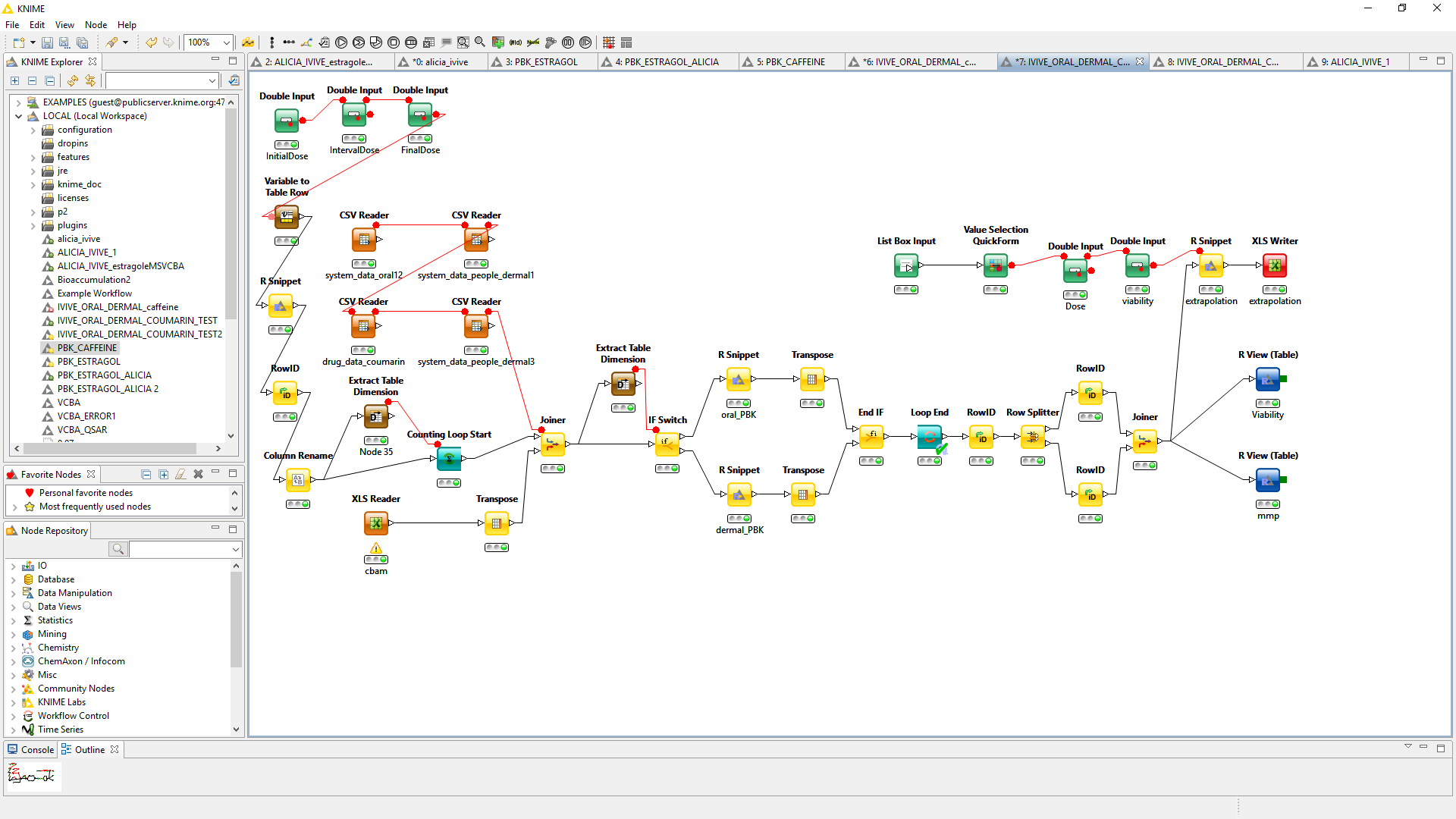
B

**Figure SM2.** The extrapolation of cell viability to external dose using Physiologically Based Kinetic Models for oral and dermal exposure.


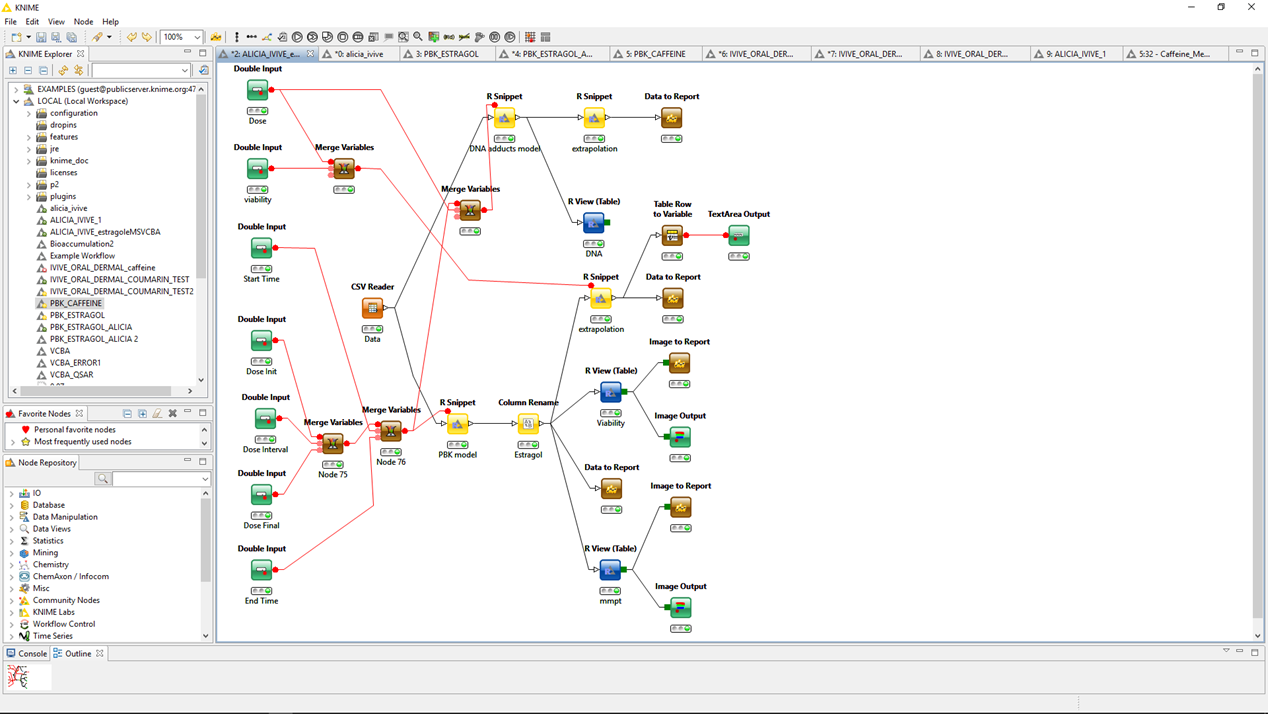


**Figure SM3.** Example of KNIME workflow for in vitro to in vivo extrapolation.


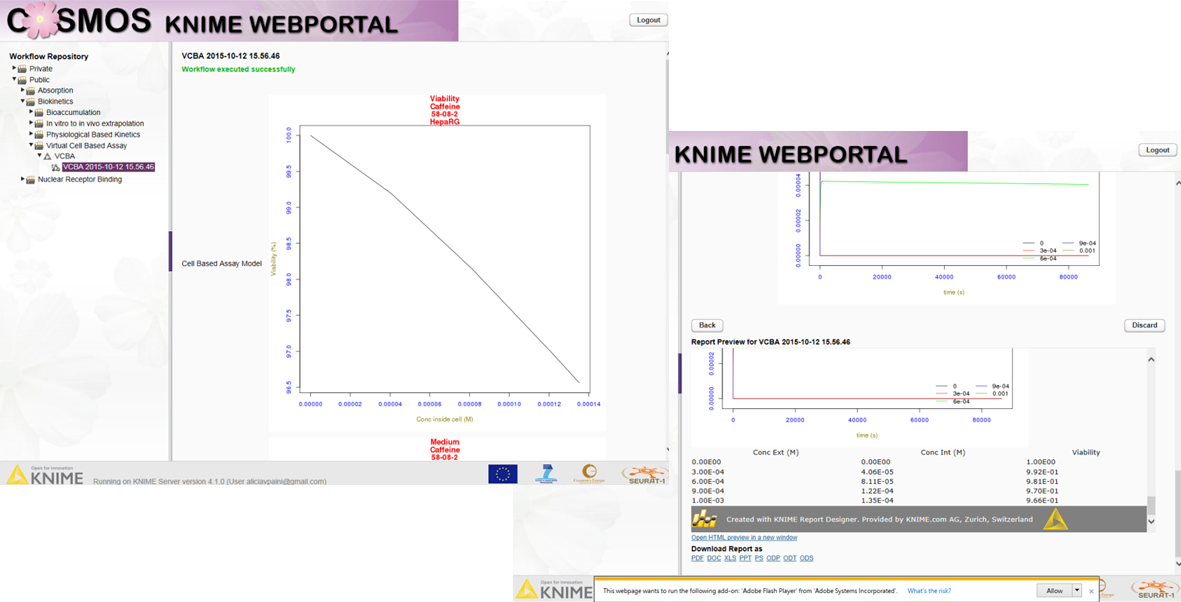


**Figure SM4.** Print screen of the VCBA output - simulations run using the COSMOS KNIME WebPortal (HepaRG cell line exposed once to caffeine), if you scroll down you can visualize also a table reporting external concentration, internal concentration and viability.
